# Synergistic Regulation of Strength and pH in Red Mud-Based Backfill Materials by CaCl_2_: Hydration, Phase Transformation and Microstructural Evolution

**DOI:** 10.3390/ma19194187

**Published:** 2026-09-30

**Authors:** Junqi Deng, Jie Wang, Jiarui Gu, Xiaoming Liu, Zengqi Zhang, Yinming Sun

**Affiliations:** 1 School of Metallurgical and Ecological Engineering, University of Science and Technology Beijing, Beijing 100083, China; d18214204593@163.com (J.D.); d202210142@xs.ustb.edu.cn (J.W.); gjr18832313078@126.com (J.G.); 2 State Key Laboratory of Advanced Metallurgy, University of Science and Technology Beijing, Beijing 100083, China

**Keywords:** calcium chloride, red mud, pH evolution, mechanical properties, underlying mechanisms

## Abstract

The high alkalinity and poor early reactivity of Bayer red mud hinder its large-scale application in mine backfill systems. Red mud-based backfill materials (RBMs) were prepared in this study using untreated red mud and other raw materials with CaCl_2_ (1–3 wt.%) added to investigate the mechanical properties, aqueous extract pH, and microstructural evolution. The results show that CaCl_2_ enhances the early age strength of RBMs. Specifically, with 1–3 wt.% CaCl_2_, the UCS of the samples at 7 d reached 5.63–6.19 MPa, a 24.0–36.3% increase over the reference (4.54 MPa). Moreover, the aqueous extract pH decreased with increasing CaCl_2_ content, reaching 10.49 at 3 wt.% CaCl_2_ after 28 d, 0.61 pH units lower than the reference. Additionally, an optimal content of CaCl_2_ promotes hydration product formation and pore structure refinement. Excessive CaCl_2_ was associated with pore structure deterioration and reduced mechanical performance at 28 d. Without the cumbersome pretreatment of raw materials, this study achieves synergistic regulation of mechanical properties and aqueous extract pH, providing a theoretical reference and technical support for the resource utilization of red mud.

## 1. Introduction

Red mud (RM), a major byproduct of alumina production from bauxite, represents a significant industrial solid waste challenge. It is estimated that the global accumulation of RM has exceeded 4 billion tons, making the development of effective disposal strategies an urgent matter [1,2]. Consequently, extensive research has focused on the valorization of RM [3,4], particularly in the development of RM-based cementitious materials as backfill materials, which offer a high-volume pathway for waste consumption [5].

Backfill technology is a critical mining approach that enhances extraction efficiency, mitigates surface solid waste accumulation, and supports sustainable resource development [6]. While the utilization of RM-based binders as backfill materials has yielded promising results, significant environmental risks remain during long-term service. Specifically, contact between the backfill body and groundwater can generate pore leachate with a pH as high as 12 [7]. The migration of this highly alkaline plume can induce severe pollution in the surrounding hydrogeological environment. Furthermore, the interaction between high-pH leachate and groundwater calcium or magnesium ions precipitates insoluble compounds, such as calcium carbonate and magnesium hydroxide. This precipitation can clog aquifer pores, thereby altering local groundwater flow fields [8]. Additionally, the leaching behavior of hazardous elements is closely governed by pH [9]. For instance, the leaching concentration of Ba^2+^ stabilizes at minimal levels near pH 10.5 [10], while Pb^2+^ exhibits its lowest leaching rate around pH 10.2 [11].

Beyond environmental concerns, previous studies indicate that elevated pH levels in RM-based materials promote the formation of interconnected coarse pore networks, which significantly compromise matrix strength [12]. In composite backfilling systems incorporating RM with coarse aggregates such as coal gangue and tailings, a wall effect causes high concentrations of Na^+^ and OH^−^ to accumulate at the aggregate surfaces. This localized enrichment induces the formation of loosely structured, preferentially oriented layers of calcium hydroxide or zeolite-like phases, creating a weak interfacial zone [13]. As a result, the bonding between the slurry and the aggregates deteriorates, and the backfill body evolves into a plate-like structure analogous to that observed in geological formations. Such structural alterations pose irreversible risks to the long-term stability and safety of mine support systems.

The alkalinity of pore water in cementitious materials is primarily controlled by the Ca(OH)_2_ content [14]. Current research on low-pH cementitious materials typically follows two strategies. The first involves selecting alternative low-alkali cements, such as sulfoaluminate, phosphate, or magnesium oxychloride cements. However, this approach is controversial due to the lack of long-term engineering validation and the high cement consumption required, which contradicts the goal of waste reduction [15,16]. The second strategy involves incorporating mineral admixtures, such as slag or fly ash, to consume Ca(OH)_2_. This approach is generally preferred as it simultaneously utilizes multiple solid waste streams, reduces cement content, and lowers leachate pH while maintaining material performance [17,18].

Recently, inorganic salt additives have emerged as an efficient strategy to modify material properties. CaCl_2_ is particularly notable for its ability to control setting times and accelerate hydration [19,20]. Preliminary observations suggest that small CaCl_2_ additions can reduce pH, but the specific pH evolution in solid waste systems remains underexplored [21]. Furthermore, the relationships among strength development at different curing ages, aqueous extract pH, and the accompanying phase and microstructural evolution with CaCl_2_ content remain insufficiently understood. Therefore, this study investigates RM-based backfill materials (RBMs) modified with CaCl_2_. Using a suite of characterization techniques, this study systematically investigated the mechanical performance, aqueous extract pH, hydration and phase transformation, and microstructural evolution of RBMs. This work aims to clarify the relationships among these changes with increasing CaCl_2_ content without resorting to cumbersome raw material pretreatment processes, providing a theoretical reference for the sustainable resource utilization of RM.

## 2. Materials and Methods

### 2.1. Experimental Materials

**Figure 1:**
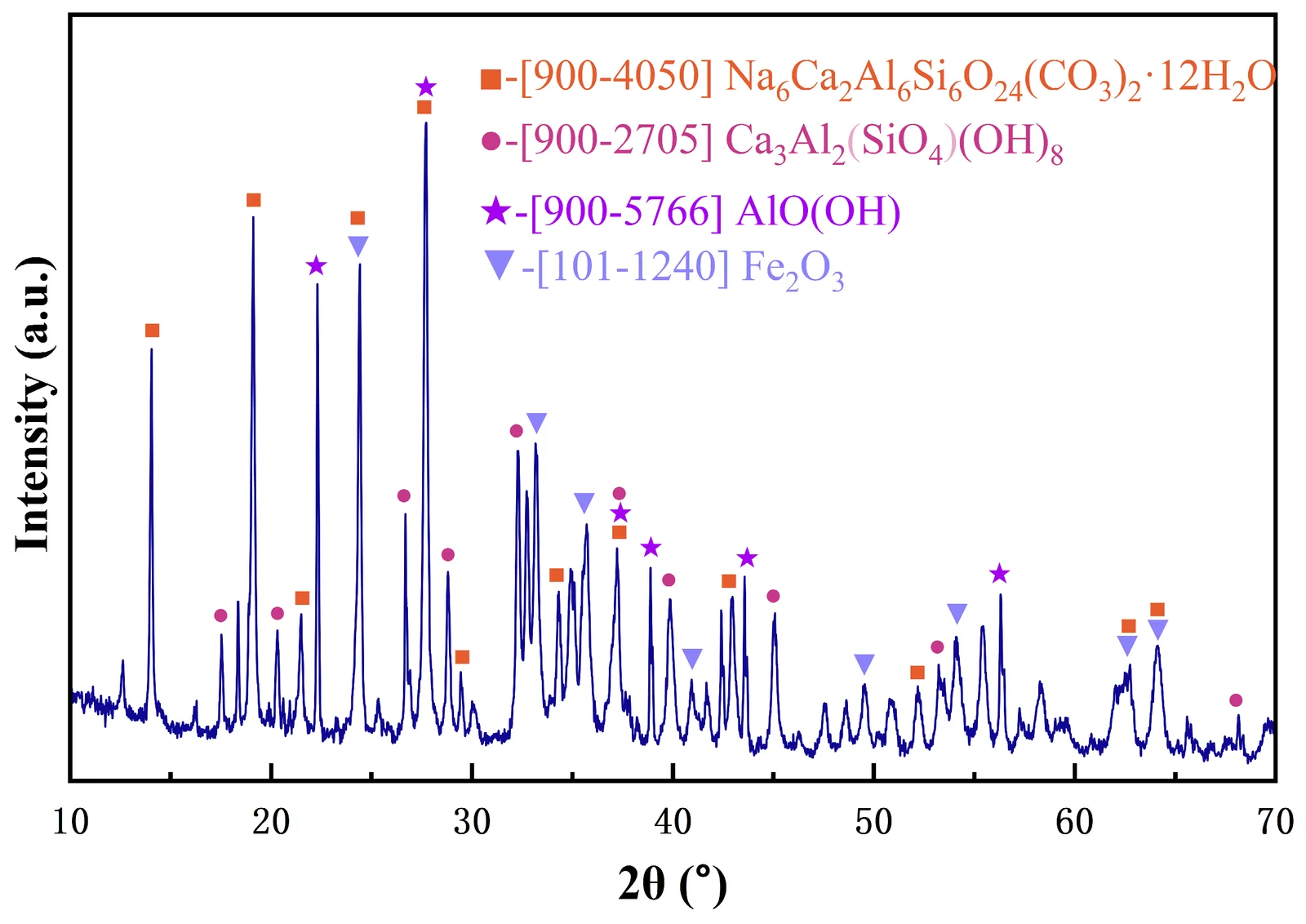
XRD pattern of the RM.

The Bayer RM used in the experiment was obtained from Shanxi Province, China. Table 1 presents the main chemical composition of the raw materials used in this experiment. As shown in the table, the chemical constituents of the RM primarily include Al_2_O_3_, SiO_2_, Fe_2_O_3_, CaO, and Na_2_O. Figure 1 shows the XRD pattern of the RM. The primary phases of the RM used in this experiment are cancrinite (Na_6_Ca_2_Al_6_Si_6_O_24_(CO_3_)_2_·12H_2_O), katoite (Ca_3_Al_2_(SiO_4_)(OH)_8_), hematite (Fe_2_O_3_), and diaspore (AlO(OH)). The measured moisture content of the RM sample was 32 wt.%.

The fly ash (FA), flue gas desulfurization gypsum (FGD), and ground granulated blast furnace slag (GGBS) used in this study were sourced from Zhongke Hongyuan Technology Co., Ltd. (Lvliang, China). FA, FGD gypsum, and GGBS were used at fixed proportions in all mixtures to form the complex binder and enhance the overall reactivity of the binder system, but their individual proportions cannot be disclosed due to commercial confidentiality. Commercial P.O.42.5 Portland cement was used. Liquid additives (LAs) consist of organic polymers used to improve the flowability of the fresh RBM slurry. To ensure calcium chloride purity, CaCl_2_ (>99%, wt.%) purchased from China National Pharmaceutical Group Chemical Reagent Co., Ltd. (Shanghai, China)was used in the study.

### 2.2. Sample Preparation

**Figure 2:**
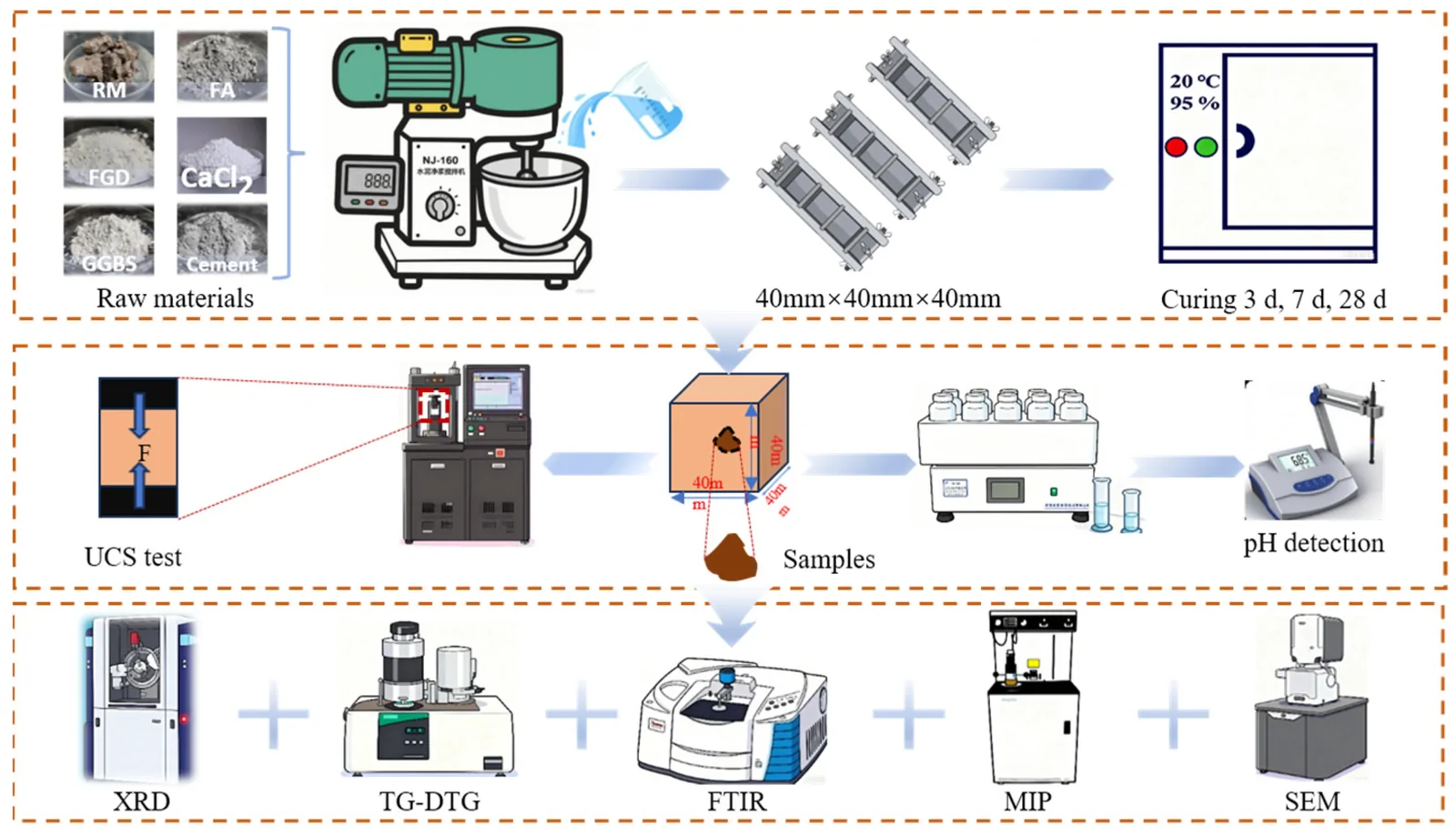
Flow chart of sample preparation and experiments.

The sample preparation procedure was as follows: All ingredients were weighed according to the specified ratios, and then poured into an agitator (370 W, NJ-160) produced by Maifang Instrument Equipment Co., Ltd. (Wuxi, China). The calculated amount of mixing water and the prescribed amount of LAs were then added simultaneously to the agitator before mechanical mixing. The same LA dosage of 0.4 wt.% relative to the total dry mass of the RBM mixture was used for all mixtures, with their liquid content excluded from the W/S calculation. During mixing, the mixing blade simultaneously rotated around its own axis (self-rotation) and revolved around the central axis of the mixing bowl (revolution). The mixture was stirred at 140 ± 5 rpm for self-rotation and 62 ± 5 rpm for revolution for 150 s. Then, stirring was continued at 285 ± 5 rpm for self-rotation and 125 ± 5 rpm for revolution for another 150 s. In this step, the mixing time was longer than the preset 2 min to ensure that CaCl_2_ was thoroughly blended with the slurry and prevent uneven distribution. The thoroughly mixed slurry was poured into a standard mold measuring 40 mm × 40 mm × 40 mm, then placed in a curing box at 20 ± 1 °C and 95 ± 1% relative humidity. The samples were demolded after 24 h of curing, and the samples were cured for 3 d, 7 d, and 28 d. Following the curing process, samples at each of these ages were used for subsequent tests. The samples used in the experiment are shown in Table 2. The sample preparation process and subsequent experimental procedures are shown in Figure 2.

### 2.3. Experimental Methods

#### 2.3.1. Unconfined Compressive Strength (UCS)

After the samples reached the designated curing ages, they were subjected to UCS testing. In the experiment, a DYE-300 microcomputer-controlled automatic press manufactured by Zhongjiao Jianyi Technology Development Co., Ltd. (Beijing, China) was used to test the UCS of the cementitious materials. To ensure the accuracy of the test, all tests were repeated three times, and the average value was taken as the final result.

#### 2.3.2. pH Detection

At each curing age, representative fragments were collected from the core regions of the three specimens used for UCS testing and combined to form one composite sample for each mixture. Crushed samples (≤3 mm) were placed into extraction bottles. Deionized water was added at a liquid-to-solid ratio of 10:1 on the basis of the moisture content of the sample. The bottles were securely capped and mounted vertically on a horizontal shaker manufactured by Bona Technology Co., Ltd. (Hangzhou, China). The shaking frequency was adjusted to 110 ± 10 cycles per minute, and the amplitude was adjusted to 40 mm. The mixture was shaken at room temperature for 8 h, and then the extraction bottles were removed and allowed to settle for 16 h. Finally, the supernatant was decanted, and the pH of the leachate was determined with a FE28 benchtop acidity meter manufactured by METTLER TOLEDO (Greifensee, Switzerland). The pH meter was calibrated using standard buffer solutions of pH 4.01, 6.86, and 9.18 before measurement.

#### 2.3.3. XRD Analysis

The equipment used for the study was a D8 ADVANCE X-ray diffractometer (Bruker, Karlsruhe, Germany) with Cu target; wavelength, 1.5406 Å; X-ray tube voltage, 40 kV; current, 40 mA. Continuous scanning mode was employed with a scan angle range of 10–90° and a scan rate of 10°/min. The samples were placed in a 40 °C oven for 24 h of drying, followed by grinding to refine the particles before testing.

#### 2.3.4. TG–DTG Analysis

The study employed a NETZSCH STA 449 F3 thermal analyser (NETZSCH-Gerätebau GmbH, Selb, Bavaria, Germany). The detection temperature range spanned from room temperature to 1000 °C at a constant rate of 10 °C/min under a nitrogen atmosphere.

#### 2.3.5. FTIR Analysis

FTIR spectra were collected using a Nicolet IS 10 infrared spectrometer from Nicolet, (Madison, WI, USA), with a wavenumber detection range of 400–4000 cm^−1^, a resolution of 4 cm^−1^, and a signal-to-noise ratio of 50,000:1.

#### 2.3.6. MIP Analysis

The pore size distribution of RBMs was tested via an Autopore IV 9500 mercury intrusion porosimeter (Micromeritics Instrument Corp., Norcross, GA, USA). Before testing, samples were cut into small pieces (approximately 5 mm in size), immersed in anhydrous ethanol, and then dried at 105 °C for 4–5 h.

#### 2.3.7. SEM Analysis

Samples were collected from the specimen cores, immersed in anhydrous ethanol for 24 h to stop hydration, and vacuum-dried at 40 °C for 24 h. After gold sputtering, the microstructures were observed using a Thermo Fisher Apreo C field-emission SEM (Thermo Fisher, Waltham, MA, USA).

## 3. Results and Discussion

### 3.1. Mechanical Properties

**Figure 3:**
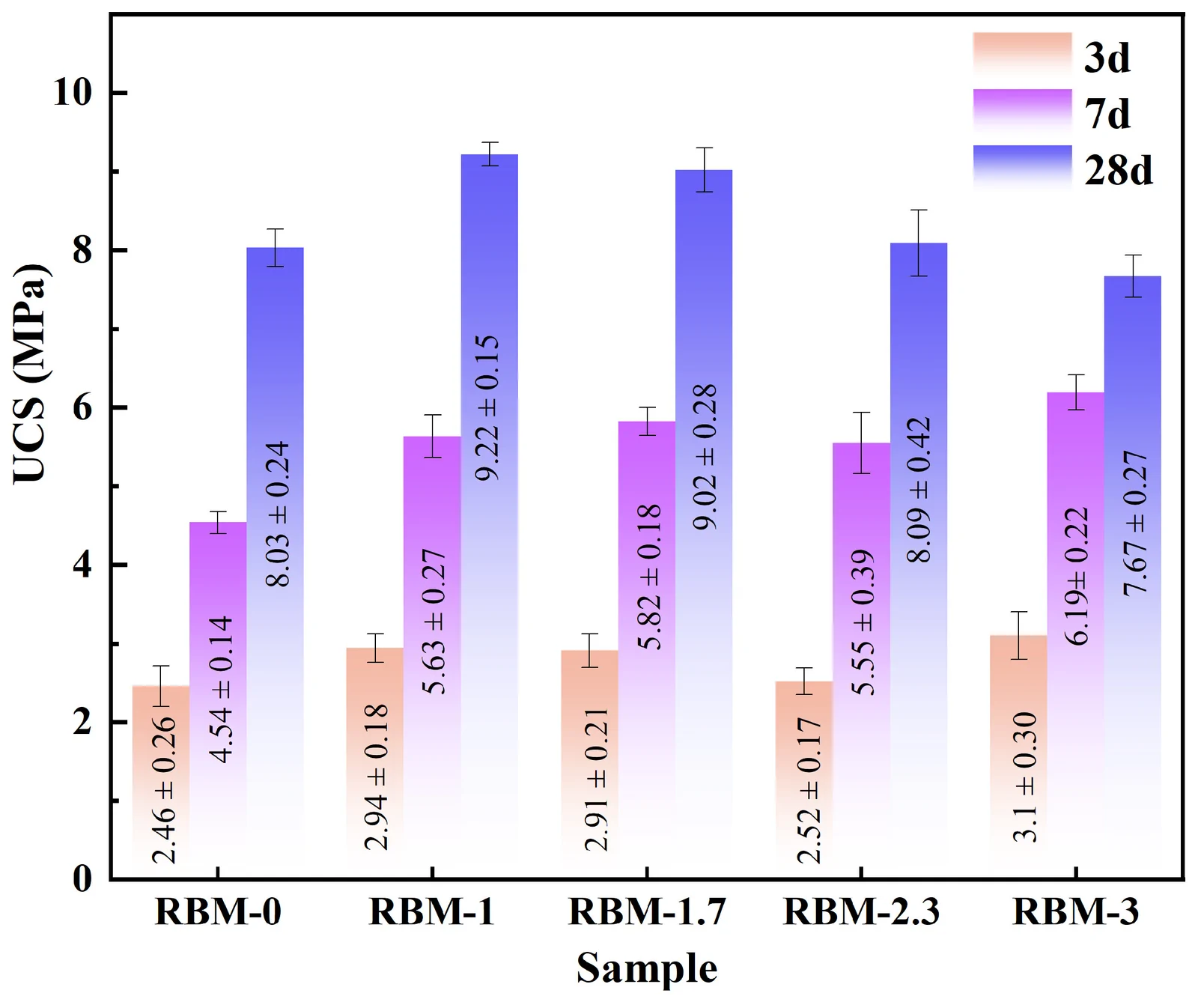
The unconfined compressive strength image of RBMs.

The specific effects of CaCl_2_ on the mechanical properties of RBMs at different ages are shown in Figure 3. At 3 and 7 d of curing, the UCS of RBMs exhibited a fluctuating upward trend with increasing CaCl_2_ content. Notably, the sample RBM-3 exhibited the fastest strength development, with its UCS reaching 3.10 MPa at 3 d and 6.19 MPa at 7 d, representing increases of 26.0% and 36.3% over the reference sample (RBM-0), respectively. This enhancement is generally attributed to the introduction of Ca^2+^, which effectively shortens the induction period, promotes the nucleation of Ca(OH)_2_ and C-S-H, and increases the hydration rate [22]. Moreover, Cl^−^ can react with tricalcium aluminate (C_3_A) during the hydration process to form Friedel’s salt (3CaO Al_2_O_3_ CaCl_2_ 10H_2_O) [23]. The formation of these insoluble layered double hydroxy salts can temporarily stabilize the structure, fill pores, and increase early strength.

However, the strength development of the RBMs changed after 28 d of curing. While CaCl_2_ dosages of 1–2.3 wt.% resulted in higher mean UCS values than the reference at 28 d, increasing the dosage to 3 wt.% resulted in a 4.5% lower mean UCS than RBM-0. This adverse effect at excessive dosages is further evidenced when analyzing the strength increments from day 7 to day 28. Specifically, the reference sample gained an additional 3.49 MPa during this period. In contrast, the samples modified with 1–3 wt.% CaCl_2_ exhibited severely restricted increments, ranging from only 3.59 to 1.48 MPa. This phenomenon indicates that while the accelerated early hydration induced by CaCl_2_ promotes initial strength development, the subsequent overproduction of specific hydrated phases distinctly inhibits late-age strength gain. This abnormal phenomenon suggests a unique microstructural influence of CaCl_2_ on the mechanical properties of RBMs. The underlying mechanisms will be investigated in subsequent sections.

### 3.2. pH Detection

**Figure 4:**
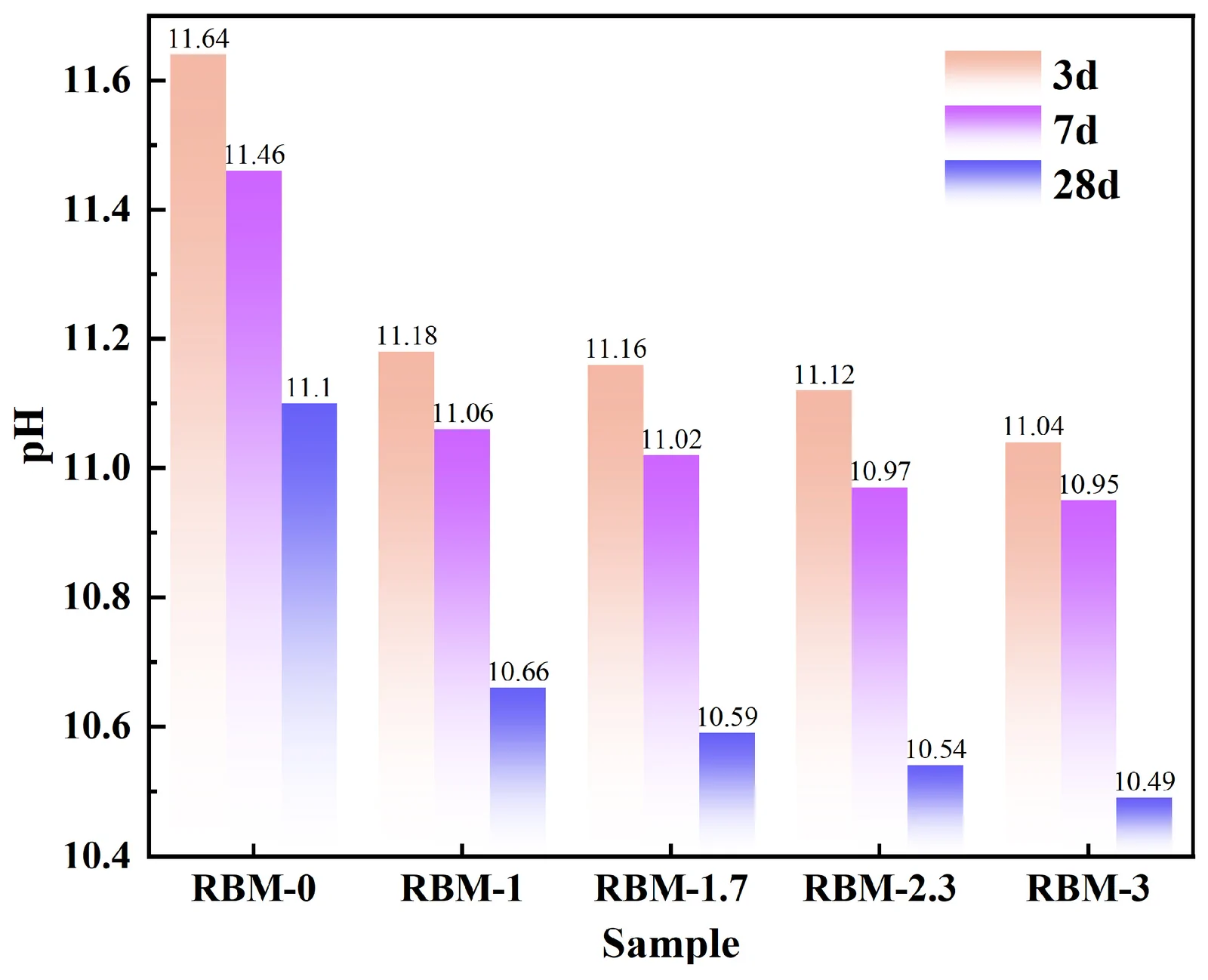
Aqueous extract pH values of RBMs at different curing ages.

Figure 4 shows the aqueous extract pH values of RBMs with various CaCl_2_ contents and specific curing periods. The evolutionary trends of all samples are highly consistent. Regardless of the CaCl_2_ content, the aqueous extract pH consistently declines with prolonged curing, indicating that the ongoing hydration process continuously consumes alkaline components. Notably, the main difference associated with CaCl_2_ addition was already apparent at 3 d. For example, at 3 d of curing, the aqueous extract pH of RBM-3 was 0.60 pH units lower than that of RBM-0. Notably, this pH differential remained nearly constant (0.61 units) even at 28 d of curing. This trend indicates that the pH evolution effect of CaCl_2_ on RBMs primarily occurs during the initial stage of hydration and hardening. Furthermore, the previous analysis of mechanical properties revealed that CaCl_2_ primarily enhances early hydration. This further suggests a possible relationship between hydration development and the observed pH evolution. Simultaneously, the aqueous extract pH decreased with increasing CaCl_2_ content. After curing for 28 d, the pH value of RBMs decreased from 11.10 to 10.49 with increasing CaCl_2_ content.

Based on the above phenomena, a preliminary analysis can be performed. First, the introduced Ca^2+^ may react with free anions in the pore solution to generate bound products like CaCO_3_ and Ca(OH)_2_, as shown in Equations (1) and (2). This direct precipitation rapidly depletes the free OH^−^ concentration, resulting in a decrease in pH. Second, the introduced Cl^−^ can participate in the formation of chloride-bearing aluminate hydrates such as Friedel’s salt, as represented schematically in Equation (3), providing a possible pathway that may contribute to OH^−^ consumption. The precise formation of these phases and the exploration of deeper, underlying mineralogical transformations are further discussed together with the subsequent solid-phase characterization results.

(1) $${\mathrm{Ca}}^{2+}{+\mathrm{CO}}_{3}^{2-}\to {\mathrm{CaCO}}_{3}↓$$

(2) $${\mathrm{Ca}}^{2+}{+2\mathrm{OH}}^{-}\to {\mathrm{Ca}(\mathrm{OH})}_{2}↓$$

(3) $${4\mathrm{Ca}}^{2+}{+2\mathrm{Al}[(\mathrm{OH})}_{4}{]}^{-}{+2\mathrm{Cl}}^{-}{+2\mathrm{OH}}^{-}{+4H}_{2}O\to {3\mathrm{CaO}\cdot \mathrm{Al}}_{2}O_{3}{\cdot \mathrm{CaCl}}_{2}{\cdot 10H}_{2}O$$

### 3.3. XRD Analysis

**Figure 5:**
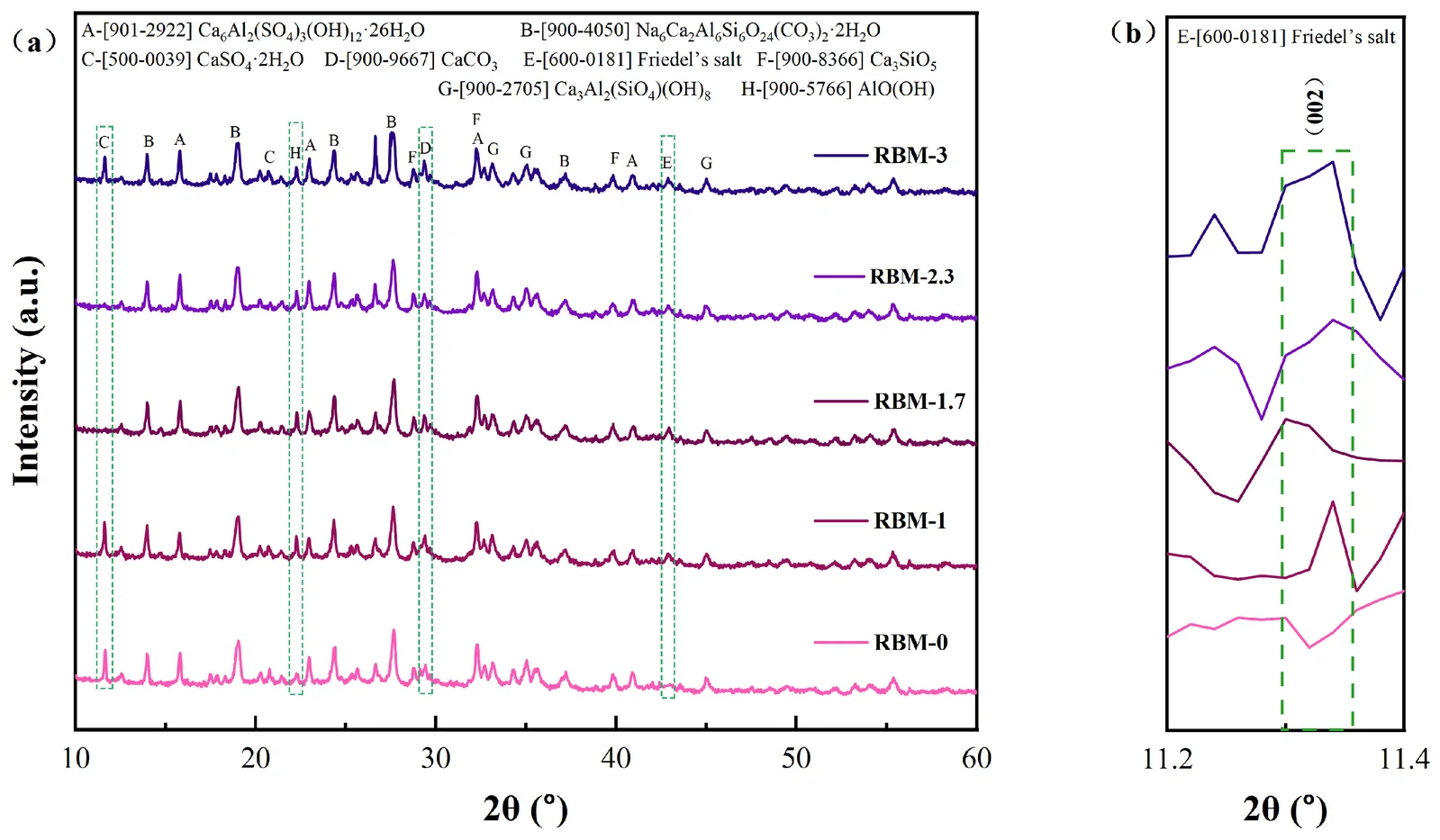
(**a**) XRD pattern of RBMs after 28 d of curing; (**b**) Locally magnified XRD pattern in the 2θ range of 11.2–11.4°.

The XRD patterns of the samples after 28 d of curing are shown in Figure 5. All samples exhibited broadly similar diffraction profiles. In addition to the phases inherited from the raw materials, the newly formed phases in the RBMs mainly include ettringite (AFt, Ca_6_Al_2_(SO_4_)_3_(OH)_12_·26H_2_O), calcite (CaCO_3_), and chloride-bearing AFm phases consistent with Friedel’s salt. The characteristic peak of AFt is primarily observed at 15.8° and 22.9° [24], which usually originates from early-stage hydration products. The characteristic peaks of Friedel’s salt appear at 11.3° and 42.88° and are generally associated with chloride binding [25]. Moreover, the typical diffraction peak of CaCO_3_ is centered at 29.34°, likely resulting from carbonation reactions or carbonate components in the raw materials [26]. Furthermore, a broad diffuse hump is evident within the 2θ range of 25–35°, which is typical of amorphous materials [27]. This indicates the formation of an amorphous C-S-H gel and a C-A-S-H gel.

Moreover, with increasing CaCl_2_ content, the peak intensity of CaSO_4_·2H_2_O at 2θ = 11.6° and 20.8° first weakens or even disappears and then reappears. In Equations (4) and (5), aluminate ions (AlO_2_^−^) react with OH^−^ to form [Al(OH)_4_]^−^, which further reacts with Ca^2+^ and SO_4_^2−^ to produce AFt. The presence of CaSO_4_·2H_2_O promotes AFt formation, whereas the introduction of additional Ca^2+^ further accelerates this reaction while consuming SO_4_^2−^ from the FGD [28]. However, the reduced SO_4_^2−^ concentration may induce the conversion of AFt to monosulfide-type calcium aluminate hydrate (AFm), thereby affecting the macroscopic properties of the material [29]. At higher CaCl_2_ contents, a transformation in the phase assemblage is observed. The high concentration of Cl^−^ triggers an anion exchange, where Cl^−^ substitutes for SO_4_^2−^ in the aluminate phases (AFt or AFm) to form the more thermodynamically stable Friedel’s salt [30]. Consequently, substantial SO_4_^2−^ is released back into the pore solution, causing the ionic product of Ca^2+^ and SO_4_^2−^ to exceed the solubility product (K_SP_) of gypsum, thereby triggering the secondary precipitation of CaSO_4_·2H_2_O [31]. At the same time, the amount of AFt in the gelling system no longer increases.

(4) $${\mathrm{AlO}}_{2}^{-}{+2H}_{2}O\to {{\left[\mathrm{Al}(\mathrm{OH}\right)}_{4}]}^{-}$$

(5) $${6\mathrm{Ca}}^{2+}{+3\mathrm{SO}}_{4}^{2-}{+2[\mathrm{Al}(\mathrm{OH})}_{4}{]}^{-}+4OH^{-}{+26H}_{2}{O=\mathrm{Ca}}_{6}{\mathrm{Al}}_{2}{{(\mathrm{SO}}_{4})}_{3}{\left(\mathrm{OH}\right)}_{12}{\cdot 26H}_{2}O$$

New peaks at approximately 11.3° and 42.88° emerged after CaCl_2_ addition. The relatively weak intensity of these reflections is likely associated with the limited abundance of Friedel’s salt and, particularly for the low-angle reflection, partial overlap with the neighboring gypsum peak [32]. The occurrence of these features only in the CaCl_2_-containing samples is also consistent with the involvement of Cl^−^ in the formation of chloride-bearing aluminate hydrates.

Additionally, the diffraction peak at 2θ = 29.34° intensified with increasing CaCl_2_ content, suggesting increased formation of crystalline CaCO_3_. Because all samples were subjected to identical curing and sample-preparation conditions, the systematically stronger calcite reflections in the CaCl_2_-containing samples indicate that CaCl_2_ promoted overall carbonate formation under the tested conditions. This interpretation is further supported by the increased mass loss in the carbonate-decomposition region observed in the TG–DTG results. Notably, the amount of Ca(OH)_2_ available for carbonation within the system is inherently limited, originating solely from the synthetic reaction between Ca^2+^ and alkaline ions present in the system. This limitation in the precursor accounts for the relatively modest overall increase in calcium carbonate formation and corresponding diffraction peak intensity, despite the observed peak elevation. Concurrently, the intensity of the diffraction peak at 2θ = 22.28°, corresponding to hexagonal octahedral aluminum metahydroxide (AlO(OH)), is also slightly enhanced. Under the synergistic influence of Ca^2+^ and Cl^−^, the original Al(OH)_3_ undergoes a structural transformation into AlO(OH) [33].

### 3.4. TG–DTG Analysis

**Figure 6:**
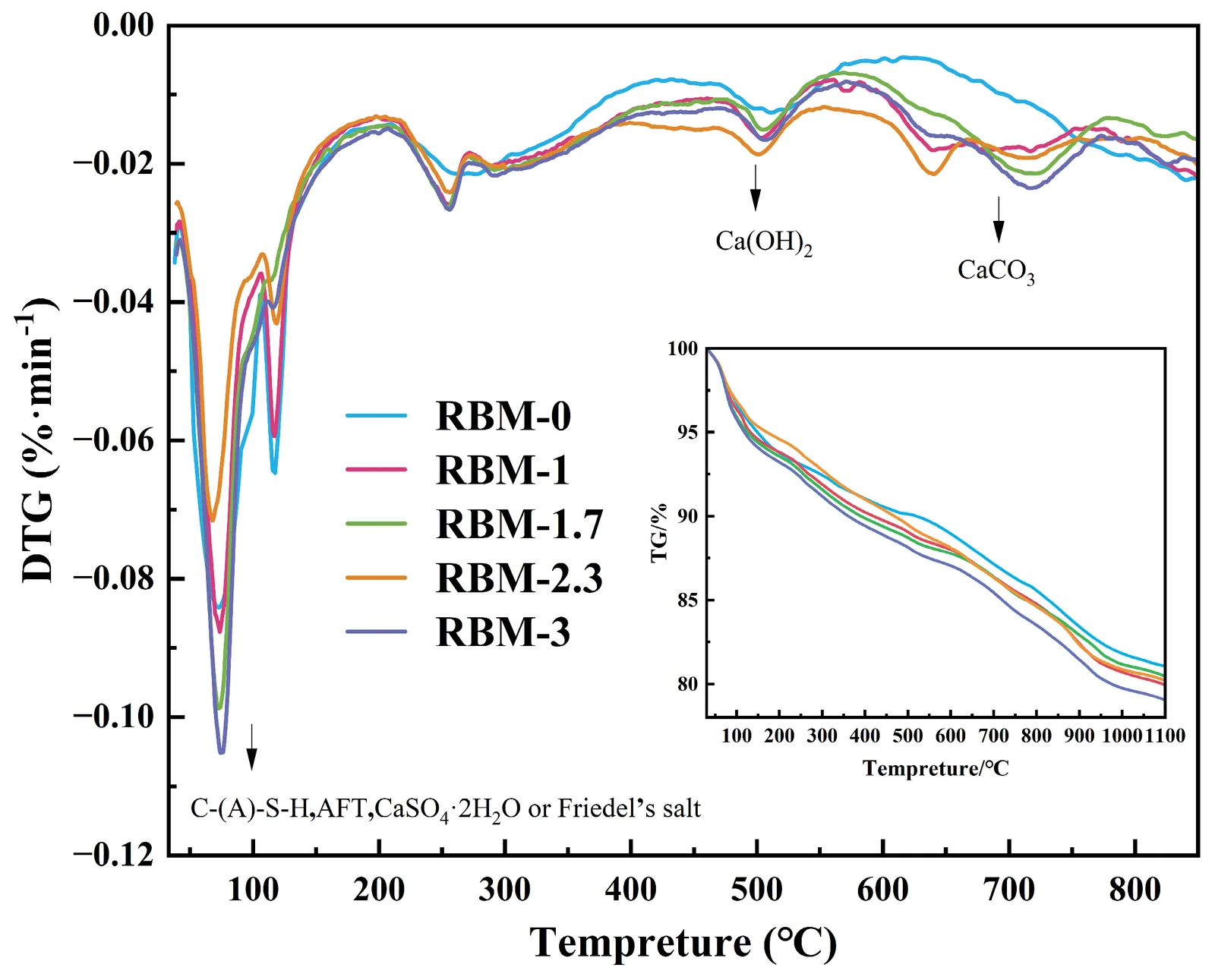
TG-DTG images of RBMs after 28 d of curing.

The TG and DTG thermograms of the samples after 28 d of curing are shown in Figure 6. As shown in the TG curve, the mass loss of the RBMs increases continuously with increasing temperature. The mass loss trends of all the samples exhibit consistent patterns under different CaCl_2_ contents. Moreover, the mass loss of the samples also increased as the CaCl_2_ content increased, indicating that the addition of CaCl_2_ promotes the formation of more hydration products. The DTG curve revealed that the thermal degradation of the samples occurs predominantly across three distinct temperature intervals: 40–200 °C, 400–570 °C, and 570–760 °C. The mass loss in the 40–200 °C range represents the loss of bound water from hydration products such as AFt and C-(A)-S-H gels, as well as the crystallization water loss from the thermal decomposition of CaSO_4_·2H_2_O to CaSO_4_·0.5H_2_O [34]. More specifically, the mass loss of bound water in the hydration products is primarily concentrated between 40 and 107 °C, whereas the crystal water loss of CaSO_4_·2H_2_O occurs mainly between 107 and 200 °C [35]. The mass loss between 400–570 °C mainly results from the thermal decomposition of Ca(OH)_2_ [36]. Finally, the loss from 570–760 °C is due primarily to the breakdown of CaCO_3_ in the RBMs [37]. A detailed analysis of these three temperature ranges, along with the effects of the CaCl_2_ content, can reveal their underlying mechanisms.

Within the temperature range of 40–200 °C, CaCl_2_ affected the two subranges differently. In the range of 40–107 °C, the rate of mass loss of the samples generally intensifies with increasing CaCl_2_ content, with the mass loss relationship being RBM-3 > RBM-1.7 > RBM-1 > RBM-0 > RBM-2.3. This trend clearly indicates that CaCl_2_ accelerates the early hydration reactions and promotes the substantial formation of gel phases, corroborating the early strength enhancements observed in Section 3.1. However, an anomalous trend is observed for RBM-2.3, which exhibits a lower mass loss rate than the reference sample. This anomaly suggests that at this specific Cl^−^ concentration, a fraction of AFt may transform into AFm, a phase inherently containing less bound water [38]. Furthermore, subtle shifts in the peak decomposition temperatures correlate directly with this mass loss behavior, a phenomenon underpinned by Cl^−^ adsorption on the AFt surfaces, which physically impedes crystal water release [39]. In the temperature range of 107–200 °C, the mass loss rate of CaSO_4_·2H_2_O shows a diphasic trend (decreasing then increasing) with increasing CaCl_2_ content, which is further confirmed by the XRD results.

TG–DTG analysis indicated that CaCl_2_ increases the mass loss from Ca(OH)_2_ decomposition within the temperature range of 400–570 °C. This trend is due to the introduction of Ca^2+^ ions and the strong hydration-promoting effect of Cl^−^ on tricalcium silicate (C_3_S) and dicalcium silicate (C_2_S), which accelerates the hydration of the RBM [40]. As a result, more Ca(OH)_2_ precipitates, leading to an increase in decomposition-related mass loss. The increased Ca(OH)_2_-related mass loss suggests that CaCl_2_ accelerated cementitious hydration and altered the assemblage of alkaline solid phases. Subsequent carbonation of these Ca-bearing phases may contribute to the lower aqueous-extract pH. However, its quantitative contribution cannot be determined from the present solid-phase characterization alone.

Finally, a clear increase in mass loss occurred within the 570–760 °C range upon the introduction of CaCl_2_, revealing enhanced carbonation of alkaline components in the system. This trend is consistent with the enhanced calcite-related diffraction intensity observed in the XRD results.

### 3.5. FTIR Analysis

**Figure 7:**
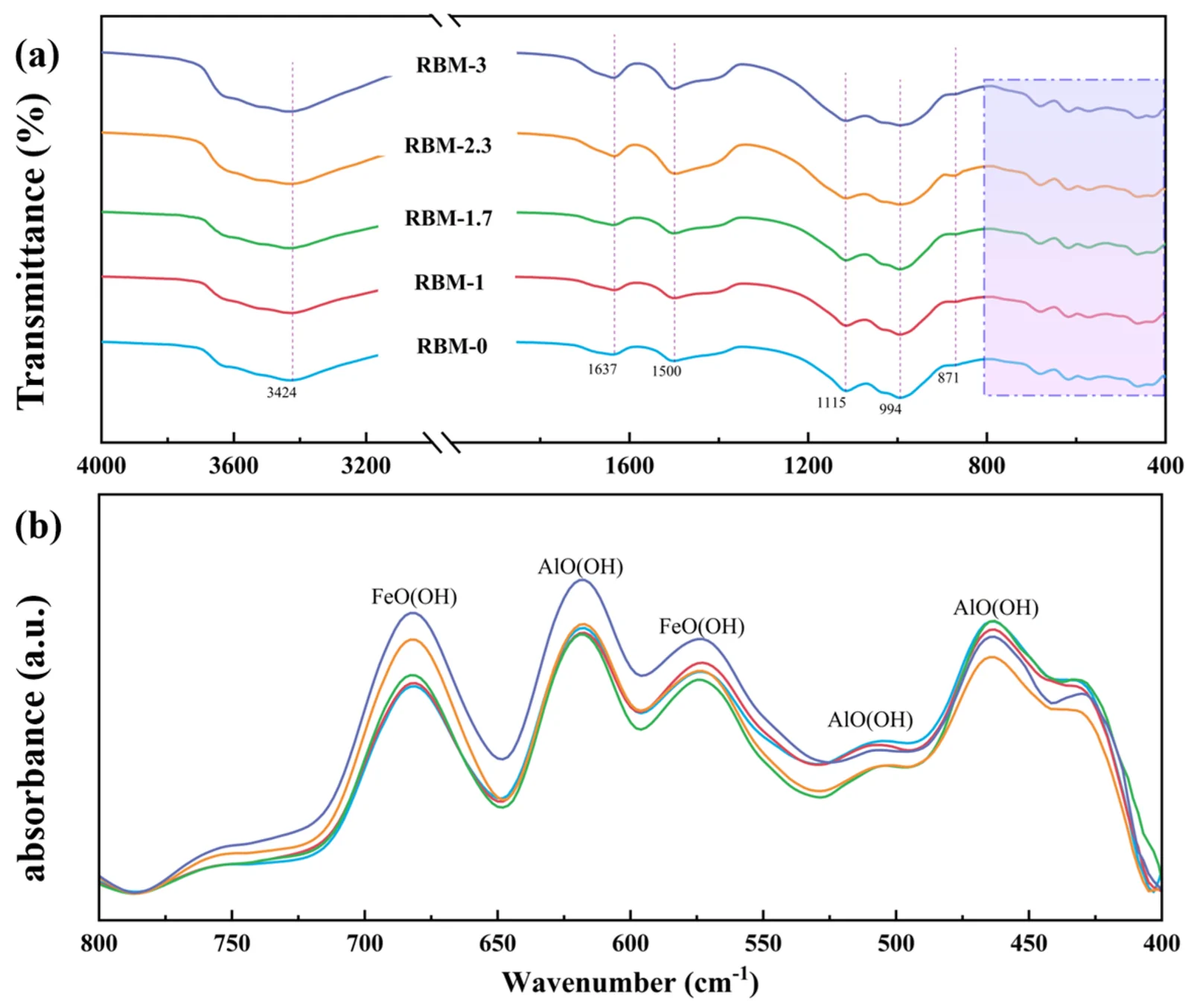
(**a**) FTIR spectra of RBMs after 28 d of curing; (**b**) Magnified view of the shaded low-wavenumber region from (**a**).

Figure 7 shows the FTIR spectrum of RBMs after 28 d of curing, which reveals its functional groups for property analysis. As shown in the FTIR spectrum, the vibration peak near 3424 cm^−1^ corresponds primarily to the stretching vibration of the -OH group [41]. The peak at 1637 cm^−1^ is attributed to the bending vibration of interlayer free water [42]. Their enhanced intensities with increasing CaCl_2_ content are consistent with a greater contribution from hydration products. The shoulder peak at 1500 cm^−1^ is attributed to the asymmetric stretching vibration of O-C-O bonds in carbonates [43], resulting from the absorption of atmospheric CO_2_ during curing. As the CaCl_2_ content increases, the morphology of this peak sharpens from a smooth shoulder into a steeper, more distinct band, which is consistent with the greater carbonate formation indicated by the XRD and TG–DTG results. The absorption peak at 1115 cm^−1^ corresponds to the asymmetric stretching vibration of SO_4_^2−^. The peak at 994 cm^−1^ belongs to the asymmetric stretching of Si-O-Si/Al linkages within the C-(A)-S-H gel network. The gradual intensification of the 994 cm^−1^ peak with higher CaCl_2_ content signifies an augmented formation of these cementitious gels [44]. Because C-(A)-S-H gel is one of the main contributors to the matrix, this spectral evidence mechanistically underpins the strength enhancements observed in the mechanical testing. The absorption peak at 871 cm^−1^, assigned to the CO_3_^2−^ in CaCO_3_ [45], reaches its maximum intensity in the RBM-2.3 and RBM-3 samples. This finding is consistent with the previous analysis results.

In the low wavenumber region (400–800 cm^−1^), several absorption bands are associated with metal–oxygen vibrations. The absorption bands near 680 cm^−1^ and 571 cm^−1^ are attributed to the overall bending vibration of FeO(OH) and the lower-frequency Fe-O stretching vibration, respectively [46]. The peaks at 617 cm^−1^, 513 cm^−1^, and 462 cm^−1^ are attributed to the stretching, twisting, and bending vibrations of Al-O, respectively [47]. These peaks are characteristic peaks of AlO(OH). These two phases are common metal oxyhydroxides in red mud-based materials [47].

### 3.6. Pore Size Distribution

**Figure 8:**
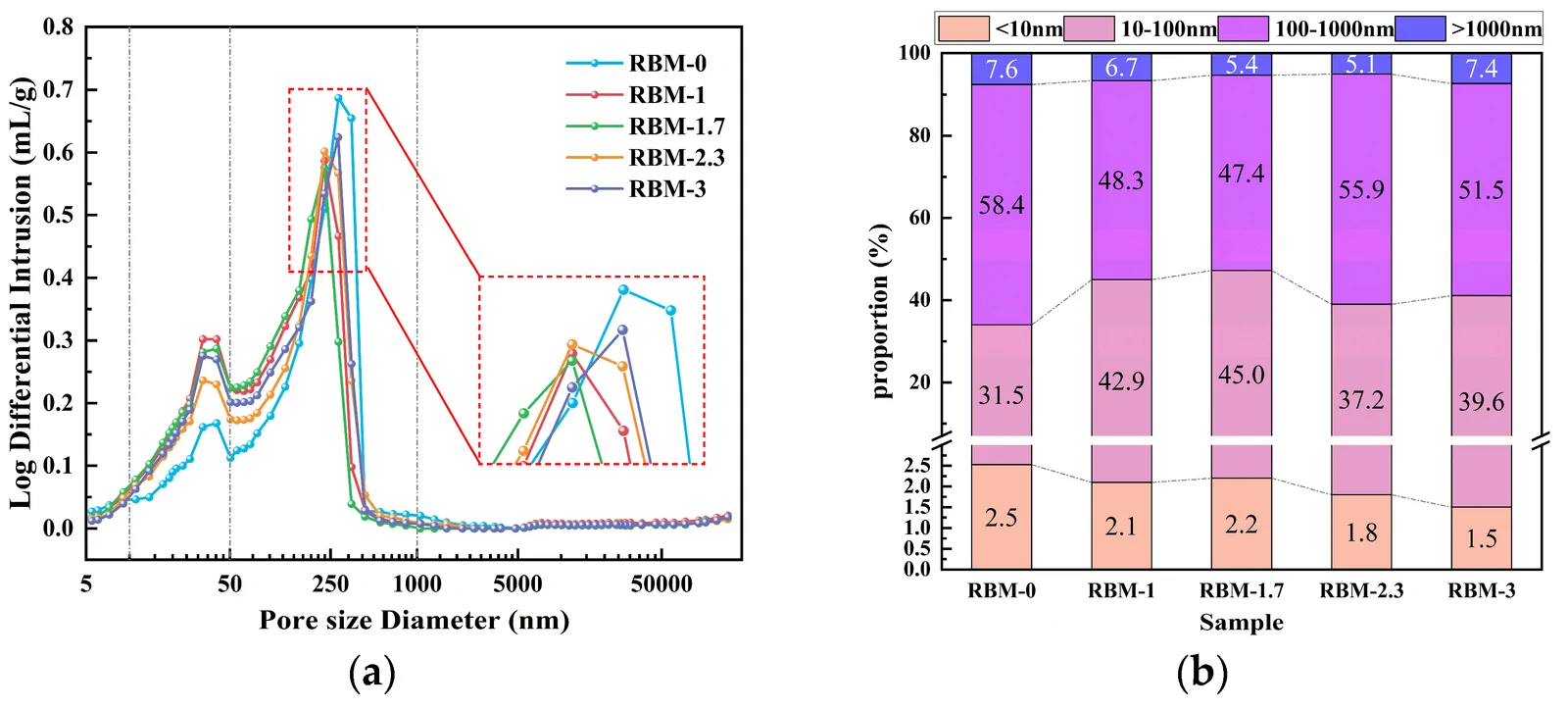
(**a**) Pore size distribution of RBMs; (**b**) Pore proportions of RBMs.

The pore structure of cementitious materials significantly influences their mechanical properties and durability. The pore structure can be categorized into four types according to pore size: ultramicropores (<10 nm), microcapillary pores (10–100 nm), macrocapillary pores (100–1000 nm), and noncapillary pores (>1000 nm). Generally, a finer pore size distribution is associated with a denser matrix structure and superior mechanical performance [48]. The pore size distributions of the RBM samples are shown in Figure 8a. The RBMs exhibited a bimodal distribution characterized by two prominent peaks centered at approximately 50 nm and 250 nm. Notably, existing studies classify pores larger than 50 nm in cement-based materials as harmful pores, as they frequently act as critical stress concentration sites that facilitate microcrack initiation under compressive loading [49]. With the addition of CaCl_2_, the peak corresponding to macrocapillary pores (approximately 250 nm) decreases in intensity and shifts toward smaller pore sizes. In contrast, the peak for microcapillary pores (approximately 50 nm) becomes more pronounced. This microstructural refinement provides direct physical evidence for the early-age UCS enhancement discussed in Section 3.1.

To further quantify the structural modification induced by CaCl_2_, an analysis of the pore volume fractions across different size ranges was conducted Figure 8b. Specifically, as the CaCl_2_ content increased, the proportion of ultramicropores in RBMs gradually decreased, whereas the proportion of noncapillary pores initially declined but subsequently rebounded at higher content. Most notably, the RBM-1.7 sample has the highest proportion of pores smaller than 100 nm, a critical size regime intrinsically linked to high matrix compactness and optimized mechanical performance. These results confirm that CaCl_2_ can only effectively suppress large pore formation and promote small pore development when its content is maintained within an optimal range. This pore structure deterioration provides strong evidence for the variation in compressive strength in the later stages discussed in Section 3.1.

### 3.7. SEM Analysis

**Figure 9:**
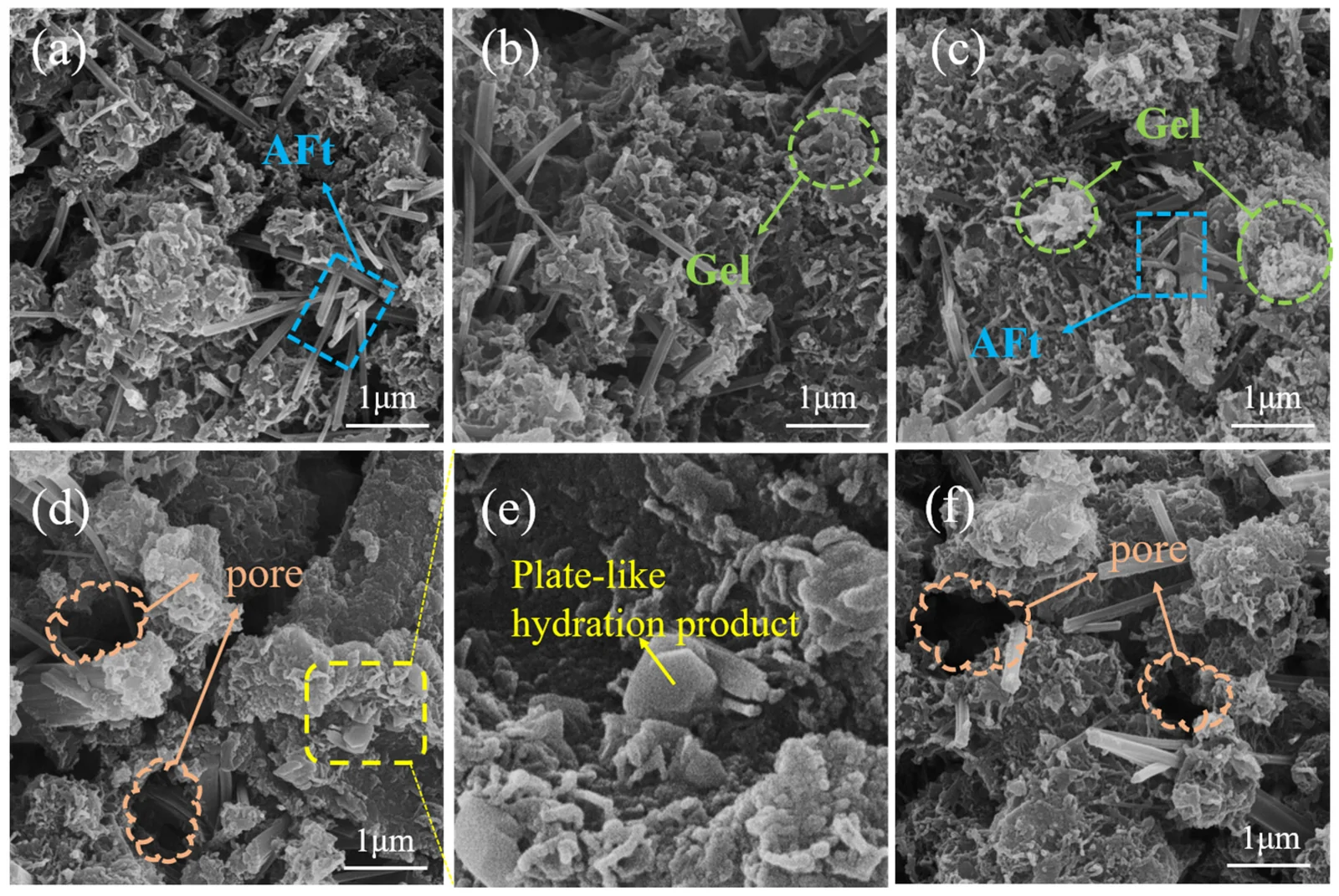
SEM images of RBMs after 28 d of curing: (**a**) RBM-0; (**b**) RBM-1; (**c**) RBM-1.7; (**d**) RBM-2.3; (**e**) Magnified view of (**d**); (**f**) RBM-3.

Figure 9 shows SEM images of RBMs after 28 d of curing. All the samples exhibited distinct needle-like and gel-like structures, regardless of the CaCl_2_ content. Based on the preceding phase analysis and previously reported morphological characteristics [50], these structures are consistent with AFt and clustered or gel-like C-(A)-S-H-type hydration products. While the compositions of different samples are similar, their microstructures differ slightly. In Figure 9a, the matrix displays a predominance of isolated AFt crystals that are inadequately bridged by the surrounding gel phases. This insufficient encapsulation results in a loosely dispersed and highly porous network. This visual lack of compactness directly corroborates the abundance of macrocapillary pores detected in the MIP analysis, fundamentally accounting for the inferior UCS of the RBM-0. In contrast, Figure 9b,c show far more compact microstructures. At these CaCl_2_ contents, the AFt crystals are densely interwoven with the adjacent C-(A)-S-H gels, effectively infilling the internal voids to construct a highly cohesive, macroscopic structural skeleton. Particularly in Figure 9c, the needle-like structures become less prominent, and the dispersed network transforms into a more complete, compact agglomerated structure. Similarly, samples RBM-1 and RBM-1.7 present fewer pores overall. This visually apparent densification is consistent with the pore size refinement observed in the MIP data.

However, when the CaCl_2_ content exceeded the optimal range, the compactness of the RBM decreased, and the pore size increased. In Figure 9d,f, although discrete cluster-like gel substances with intact structures can be observed relatively clearly in the microstructures of samples RBM-2.3 and RBM-3, more and deeper pores were observed overall. This phenomenon is attributed to the excessive hydration products and Friedel’s salt, as mentioned earlier. During the later stages of hardening, continued crystallization of hydration products within the confined pores may generate local internal stresses and disturb the established gel network. This microstructural change is consistent with the anomalous rebound in the proportion of noncapillary (>1000 nm) pores in the MIP test and may contribute to the abnormal decrease in compressive strength of RBM-3 samples at 28 d of curing.

## 4. Mechanism Discussion

An analysis based on compressive strength, pH evolution, hydration products, and microstructure revealed that adding CaCl_2_ lowered the aqueous-extract pH, promoted the formation of hydration products, and improved the microstructure of RBMs.

After the introduction of CaCl_2_ into the RBM system, the contents of primary hydration products such as AFt and C-(A)-S-H increased (Figure 5 and Figure 6). These abundant hydration products rapidly interweave within the matrix to construct a dense skeletal structure. Concurrently, Cl^−^ in the pore solution reacted with the aluminate phases to precipitate Friedel’s salt, a type of insoluble layered double hydroxide. The localized formation of Friedel’s salt may contribute to filling the internal micro-pores within the gel skeleton, leading to a substantial increase in UCS at the 3 and 7 d curing stages (Figure 3). However, in the later curing stage (28 d), excessive CaCl_2_ content led to the generation of abundant hydration products and Friedel’s salt. The continued crystallization of hydration products within the confined pore structure may generate local internal stresses and disturb the established gel network, which may contribute to the observed reduction in mechanical strength at 28 d (Figure 3).

**Figure 10:**
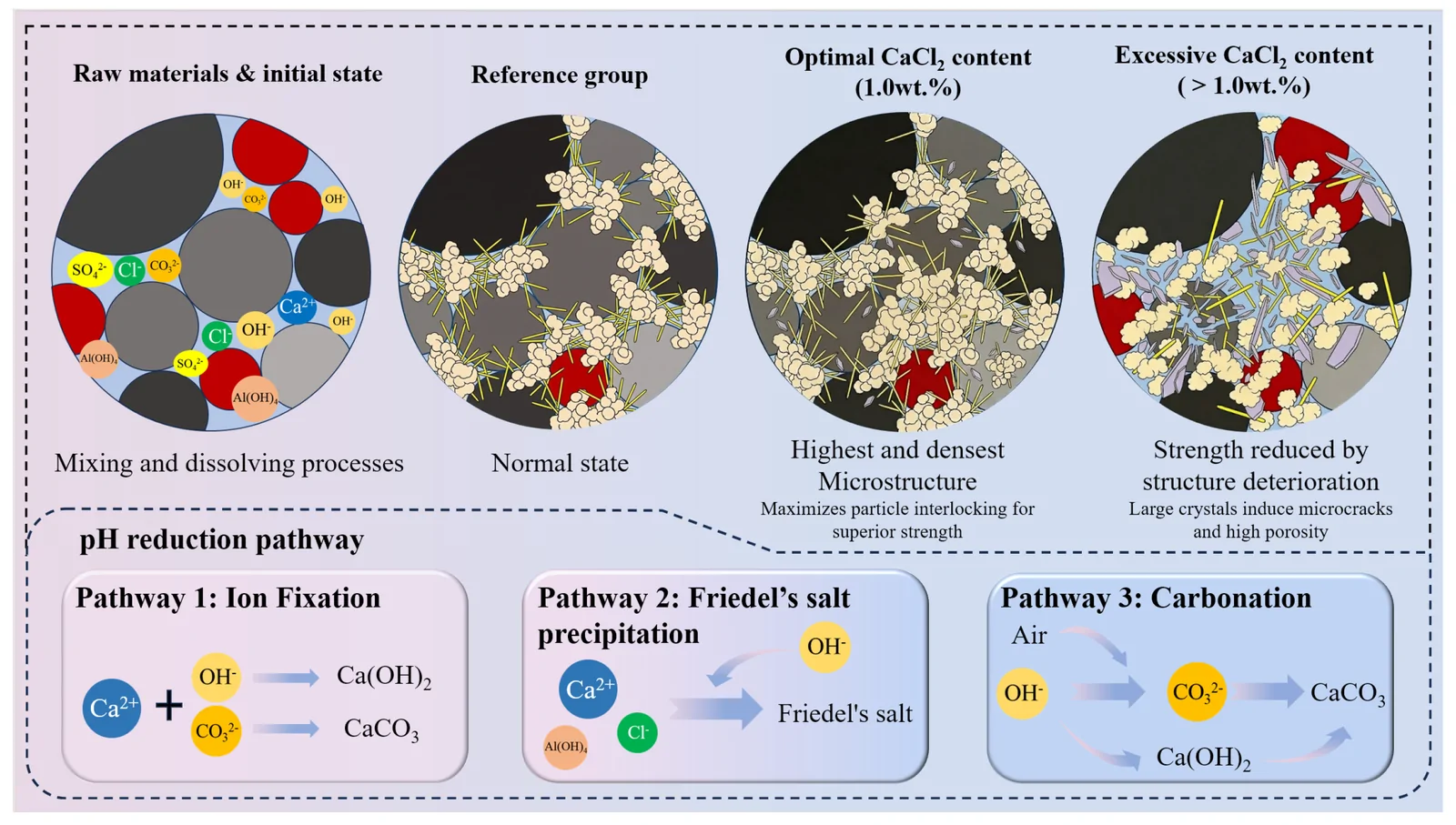
Schematic diagram of the synergistic regulation mechanism of mechanical properties and pH in RBMs.

During the continuous hydration reaction, the aqueous extract pH continuously decreases. The pH measurements show that the pH of RBMs decreases with increasing CaCl_2_ (Figure 4). In summary, the Ca^2+^ spontaneously reacts with free alkaline anions in the system to generate stable precipitates such as CaCO_3_ and Ca(OH)_2_ (Figure 5), contributing to the decrease in pH. In addition, the precipitation of Friedel’s salt involving Ca^2+^, aluminate species, Cl^−^, and OH^−^ provides a possible OH^−^-consuming pathway, which may also contribute to the reduction in pH. Furthermore, TG–DTG results showed an increase in Ca(OH)_2_-related mass loss after CaCl_2_ addition. As the most susceptible substance to carbonation in the system, the abundant generation of Ca(OH)_2_ indirectly accelerated the overall carbonation reaction (Figure 5 and Figure 6), which may also contribute to the decrease in aqueous extract pH. A simplified mechanism diagram is shown in Figure 10.

## 5. Conclusions

The effects of CaCl_2_ on the mechanical properties and aqueous extract pH of RBMs were investigated. The main findings are as follows:

(1) CaCl_2_ enhances the early UCS of RBMs. Specifically, with 1–3 wt.% CaCl_2_, the UCS of the samples at 7 d reached 5.63–6.19 MPa, a 24.0–36.3% increase over the reference (4.54 MPa). At 28 d of curing, while suitable CaCl_2_ addition further improves the strength up to 9.22 MPa, an excessive dosage (3.0 wt.%) causes a 4.5% reduction compared to the reference.
(2) The aqueous extract pH of RBMs exhibits a continuous negative correlation with CaCl_2_ content, and this pH reduction predominantly occurs during the early stage (3 d). After 28 d of curing, the aqueous extract pH decreased from 11.10 for RBM-0 to 10.49 for RBM-3, corresponding to an absolute reduction of 0.61 pH units.
(3) CaCl_2_ regulates the pH of RBMs through multiple mechanisms. The introduced Ca^2+^ immobilizes free alkaline ions via precipitation and accelerates overall carbonation. Meanwhile, Friedel’s salt precipitation may provide an additional pathway for OH^−^ consumption.
(4) Microstructurally, an optimal CaCl_2_ dosage refines the pore network of RBMs. It accelerates hydration to generate more hydration products and induces the formation of Friedel’s salt, which collectively fills internal macro-pores and enhances matrix compactness.

Overall, this study reveals a dosage-dependent relationship among the mechanical performance, aqueous extract pH, hydration and phase evolution, and microstructural development of CaCl_2_-modified RBMs. Future work should focus on longer curing periods. Additional solution chemistry and leaching tests under groundwater exposure would further clarify the long-term performance of the material.

## Figures and Tables

**Table 1:** Main chemical components of the raw materials (wt.%).

Composition	Al_2_O_3_	SiO_2_	Fe_2_O_3_	CaO	K_2_O	TiO_2_	SO_3_	Na_2_O
RM	29.34	23.43	16.01	11.48	0.98	4.39	1.07	11.34
FA	37.45	47.17	4.00	4.43	0.61	2.65	1.08	-
FGD	0.46	2.03	0.41	47.64	0.07	-	48.50	0.11
GGBS	12.92	31.42	1.01	41.74	-	1.30	3.07	-
Cement	10.00	22.50	3.87	54.01	0.89	0.58	3.75	-

**Table 2:** Raw material ratios of RBMs (wt.%).

Sample	RM ^a^	Complex Binder	Cement	CaCl_2_ ^b^	W/S
RBM-0	50	46	4	0	0.6
RBM-1	50	46	4	1.0	0.6
RBM-1.7	50	46	4	1.7	0.6
RBM-2.3	50	46	4	2.3	0.6
RBM-3	50	46	4	3.0	0.6

Note: ^a^ RM was proportioned on an as-received wet-mass basis and contained 32 wt.% moisture. ^b^ The content of CaCl_2_ is expressed as a weight percentage relative to the total mass of RBMs.

## Data Availability

The original contributions presented in this study are included in the article. Further inquiries can be directed to the corresponding authors.

## Abbreviations

The following abbreviations are used in this manuscript:

FA	Fly ash
FGD	Flue gas desulfurization gypsum
GGBS	Ground granulated blast furnace slag
LAs	Liquid additives
RBMs	Red mud-based backfill materials
RM	Red mud
UCS	Unconfined compressive strength
